# Elevated Tumor Lactate and Efflux in High-grade Prostate Cancer demonstrated by Hyperpolarized ^13^C Magnetic Resonance Spectroscopy of Prostate Tissue Slice Cultures

**DOI:** 10.3390/cancers12030537

**Published:** 2020-02-26

**Authors:** Renuka Sriram, Mark Van Criekinge, Justin DeLos Santos, Fayyaz Ahamed, Hecong Qin, Rosalie Nolley, Romelyn DeLos Santos, Z. Laura Tabatabai, Robert A. Bok, Kayvan R. Keshari, Daniel B. Vigneron, Donna M. Peehl, John Kurhanewicz

**Affiliations:** 1 Department of Radiology and Biomedical Imaging, University of California, San Francisco, CA 94158, USA; Renuka.Sriram@ucsf.edu (R.S.); mark.vancriekinge@ucsf.edu (M.V.C.); jmdeloss@gmail.com (J.D.S.); fayyaz_ahamed@berkeley.edu (F.A.); hecong.qin@ucsf.edu (H.Q.); Romelyn.DelosSantos@ucsf.edu (R.D.S.); robert.bok@ucsf.edu (R.A.B.); dan.vigneron@ucsf.edu (D.B.V.); 2 Department of Urology, Stanford University, Stanford, CA 94305, USA; tinyn@stanford.edu; 3 Department of Clinical Pathology, University of California, San Francisco, San Francisco, CA 94158, USA; Laura.Tabatabai@ucsf.edu; 4 Department of Radiology, Memorial Sloan Kettering Cancer Center, New York, NY 10065, USA; rahimikk@mskcc.org

**Keywords:** hyperpolarized ^13^C magnetic resonance (HP ^13^C MR), dynamic nuclear polarization (DNP), aerobic glycolysis, lactate dehydrogenase (LDH), lactate efflux, prostate cancer

## Abstract

Non-invasive assessment of the biological aggressiveness of prostate cancer (PCa) is needed for men with localized disease. Hyperpolarized (HP) ^13^C magnetic resonance (MR) spectroscopy is a powerful approach to image metabolism, specifically the conversion of HP [1-^13^C]pyruvate to [1-^13^C]lactate, catalyzed by lactate dehydrogenase (LDH). Significant increase in tumor lactate was measured in high-grade PCa relative to benign and low-grade cancer, suggesting that HP ^13^C MR could distinguish low-risk (Gleason score ≤3 + 4) from high-risk (Gleason score ≥4 + 3) PCa. To test this and the ability of HP ^13^C MR to detect these metabolic changes, we cultured prostate tissues in an MR-compatible bioreactor under continuous perfusion. ^31^P spectra demonstrated good viability and dynamic HP ^13^C-pyruvate MR demonstrated that high-grade PCa had significantly increased lactate efflux compared to low-grade PCa and benign prostate tissue. These metabolic differences are attributed to significantly increased *LDHA* expression and LDH activity, as well as significantly increased monocarboxylate transporter 4 (MCT4) expression in high- versus low- grade PCa. Moreover, lactate efflux, LDH activity, and MCT4 expression were not different between low-grade PCa and benign prostate tissues, indicating that these metabolic alterations are specific for high-grade disease. These distinctive metabolic alterations can be used to differentiate high-grade PCa from low-grade PCa and benign prostate tissues using clinically translatable HP [1-^13^C]pyruvate MR.

## 1. Introduction

A pressing need facing the clinical management of men with primary prostate cancer (PCa) is an accurate method for distinguishing indolent from aggressive, potentially lethal, cancer in individual patients [1]. While the American Cancer Society estimates that 174,650 men will be diagnosed with PCa in the United States in 2019, only 18% (≈31,620) of these patients will have aggressive disease resulting in death due to PCa [2]. Screening for PCa with a serum prostate-specific antigen (PSA) test has reduced mortality through identification and treatment of high-risk cancer at an earlier time-point, but at the cost of over-diagnosis and over-treatment of low-risk tumors with questionable benefit for many patients [3,4,5,6]. This dilemma reflects the biological heterogeneity of PCa and incites efforts to distinguish the small number of patients having aggressive PCa who will benefit from therapeutic intervention and the larger proportion of patients whose tumors are unlikely to progress [1,7,8,9,10,11]. Proposed solutions to this problem include screening more selectively, reserving treatment for men with aggressive cancer, and guiding those with low-risk/indolent disease to active surveillance, with serial assessment and curative treatment if there is evidence of progression [12,13]. Currently, clinical-pathological-molecular variables including serum PSA levels, biopsy characteristics (Gleason grade, volume of cancer, genetic signatures), and stage of disease all provide important insights into the risk of progression and death [14], but unfortunately fail to discriminate aggressive versus indolent cancer in many patients. While most patients can live with low-grade PCa throughout their lifespans without treatment, studies have shown that, over time, approximately one-third of these patients will be reclassified with higher-grade cancer and higher risk for progression, and will be subsequently treated [15]. The current state-of-the-art for imaging localized PCa, multiparametric ^1^H magnetic resonance imaging (MRI), has demonstrated the ability to localize tumors for subsequent biopsy and treatment, but cannot consistently grade tumor aggressiveness accurately in individual patients [16]. Therefore, there is an unmet clinical need for an accurate, non-invasive imaging method to detect aggressive PCa in men at diagnosis and during active surveillance so that timely treatment of this potentially deadly disease can be initiated only when needed.

Increasing evidence points to PCa as a disease strongly linked to abnormal metabolism, and several unique metabolic shifts have been associated with the presence and aggressiveness of PCa [17,18,19]. Significant reductions in citrate and polyamines and increased choline metabolites associated with PCa and its progression can be assessed by ^1^H magnetic resonance spectroscopic imaging (MRSI). However, changes in lactate metabolism that occur in PCa have been largely ignored due to the inability to resolve lactate from lipid in vivo by ^1^H MRSI [20].

Hyperpolarized (HP) ^13^C magnetic resonance (MR) is a powerful metabolic imaging method that uses specialized instrumentation to provide signal enhancements of over 10,000-fold for ^13^C-enriched, endogenous, non-radioactive compounds [21]. While the metabolism of PCa is often inadequately evaluated using ^18^F-fluorodeoxyglucose positron emission tomography (which assesses glucose uptake and phosphorylation) [22,23], HP ^13^C MR detects downstream metabolism, specifically the conversion of HP [1-^13^C]pyruvate to [1-^13^C]lactate catalyzed by lactate dehydrogenase (LDH). In preclinical studies, this imaging method has shown promise not only for detecting PCa but also for assessing its aggressiveness (pathologic Gleason grade) [24,25]. A single time-point ^13^C MRSI study of HP [1-^13^C]pyruvate metabolism in the Transgenic Adenocarcinoma of Mouse Prostate (TRAMP) model demonstrated a significant increase in HP [1-^13^C]lactate signal in high- versus low-grade prostate tumors [24]. In a more recent study employing a 3D dynamic dual-agent HP ^13^C MRSI approach with [1-^13^C]pyruvate and ^13^C urea, a significant increase in the rate of conversion of HP [1-^13^C]pyruvate to [1-^13^C]lactate and a significant decrease in ^13^C urea perfusion in high- versus low-grade TRAMP tumors were observed [25]. The conversion of HP [1-^13^C]pyruvate to [1-^13^C]lactate is increased in PCa due in part to genomic loss of the PTEN locus, leading to activation of the PI3K/AKT pathway, and amplification of chromosome 8q, including the MYC gene, which occurs in up to 70% and 30% of prostate cancers, respectively [26]. Additionally, studies have shown that high expression of monocarboxylate transporter 4 (MCT4), a lactate exporter essential for maintaining high levels of glycolysis and lactate production [27,28], is associated with more aggressive PCa and therapeutic resistance [29,30].

Unfortunately, murine models do not fully recapitulate the pathologic and biologic heterogeneity of PCa in patients, and therefore the metabolic changes observed with the development of aggressive PCa in the TRAMP model may not fully reflect what will be observed in patients by HP ^13^C MRSI. Prior studies have demonstrated that thin, precision-cut slices of prostate tissues obtained at radical prostatectomy and maintained in culture within a nuclear magnetic resonance (NMR)-compatible perfusion system, referred to as a tissue culture bioreactor, can provide a realistic model of the human situation with the structure, function, and metabolism of benign and malignant prostate tissue slice cultures (TSCs) recapitulating observations in patients [19,31]. Specifically, it was shown that malignant TSCs exhibited steady-state glycolytic and phospholipid metabolism and bioenergetics that recapitulated the features of PCa in vivo in patients [19].

The goal of this study is to provide the first functional evidence in living patient-derived tissues that tumor HP [1-^13^C]lactate and efflux provide accurate measures of PCa aggressiveness/pathologic grade, thereby validating prior animal studies and setting the stage for testing HP [1-^13^C]pyruvate MR imaging in patients to noninvasively assess disease aggressiveness. This was accomplished using a combination of quantitative ^1^H high resolution–magic angle spinning (HR–MAS) NMR of snap-frozen patient biopsies, and HP ^13^C NMR studies of living human prostate tissues obtained at surgery and maintained in an NMR-compatible three-dimensional (3D) tissue culture bioreactor [19].

## 2. Results

### 2.1. Gleason Grade-Dependent Increase in Lactate Concentration and LDHA Expression in Snap-Frozen Patient Biopsies

**Figure 1:**
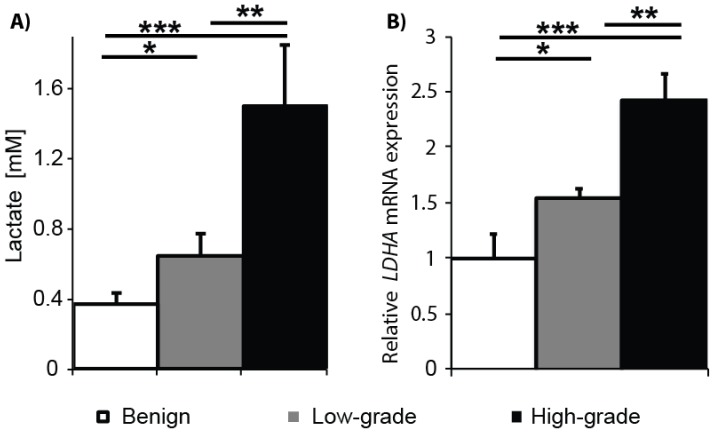
Lactate levels and lactate dehydrogenase A (*LDHA*) mRNA expression are significantly increased in cancer versus benign prostate tissues and in high- versus low-grade cancer. (**A**) Steady state lactate concentration (mM) was measured by ^1^HR-MAS in benign biopsies (N = 15), biopsies containing low-grade cancer (N = 11), and biopsies containing high-grade cancer (N = 4); (**B)** *LDHA* mRNA expression was quantified by qRT-PCR in benign, low-grade cancer, and high-grade cancer biopsies (N = 3 each). (* *p* < 0.05 and > 0.005, ** *p* < 0.005 and > 0.0005, and *** *p* < 0.0005).

To test the hypothesis that tumor tissue lactate concentrations increase in a Gleason grade-dependent fashion in PCa, lactate concentrations were measured in snap-frozen prostate biopsies using quantitative ^1^H HR–MAS NMR spectroscopy. The bar plot shown in Figure 1A summarizes the intracellular lactate concentrations in benign (N = 15), low-grade cancer (Gleason score ≤ 3 +4; N = 11), and high-grade cancer (Gleason score ≥ 4 + 3; N = 4) biopsies. Steady state lactate concentrations increased from 0.37 ± 0.06 mM in benign tissues to 0.65 ± 0.12 mM in low-grade cancer and 1.5 ± 0.35 mM in high-grade cancer. Intracellular lactate concentrations were significantly different among all three types of tissues. Relative levels of LDHA mRNA expression also significantly differed among the tissues, increasing 1.6-fold in low-grade cancer and 2.5-fold in high-grade cancer compared to benign tissue (Figure 1B). The LDH protein is a tetramer comprised of LDH-M and/or LDH-H subunits that are coded by the gene LDHA and LDHB, respectively. Despite the structural similarity, there are subtle differences in the kinetic properties that favor the formation of lactate from pyruvate by the LDH-M subunit and vice versa for the LDH-H subunit. This shift in the LDH isoform composition results in the net increase in tissue lactate concentration. Altogether, these results support the concept of an increase in glycolysis with the transition of benign prostate tissue to cancer and with the progression of cancer from low-risk (Gleason score ≤ 3 + 4) to high-risk (Gleason score ≥ 4 + 3).

### 2.2. ^31^P Spectroscopy of TSCs: Tissue Viability and Grade-Dependent ^31^P Spectral Changes

**Figure 2:**
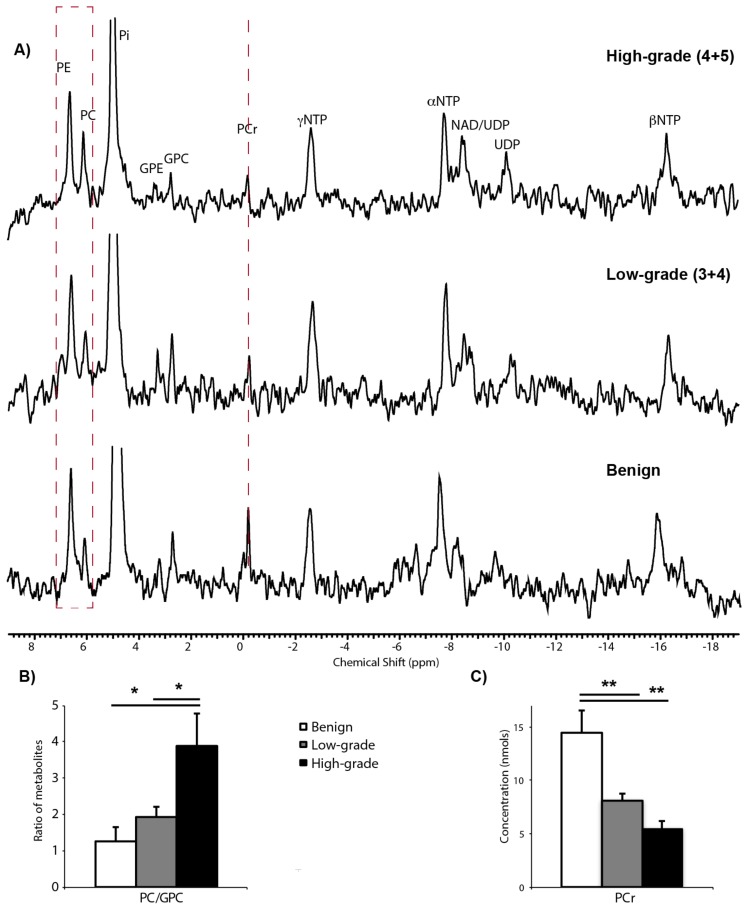
^31^P spectroscopy of TSCs in the 5-mm bioreactor. (**A**) Representative ^31^P spectra from benign prostate tissue slice culture (TSC) (bottom spectrum), TSC containing Gleason score 3 + 4 cancer (middle spectrum, 53% of the TSC composed of cancer cells) and TSC containing Gleason score 4 + 5 cancer (top spectrum, 33% of the TSC composed of cancer cells). Resonances due to phosphomonoesters [phosphocholine (PC), phosphoethanolamine (PE)], inorganic phosphate (Pi), phosphodiesters [glycerophosphocholine (GPC), glycerophosphoethanolamine (GPE), phosphocreatine (PCr)], nucleotide triphosphates (α,β,γ-NTPs), nicotinamide adenine dinucleotide (NAD), and uridine diphosphate (UDP) sugars are reproducibly visible in the ^31^P spectra of all 3 tissue types. While the α,β,γ-NTP resonance remained relatively constant between tissue types, differences were observed in the phosphomonoester and phosphocreatine regions of the ^31^P spectra (dashed red lines); (**B**) Bar graphs showing significant increases in PC/GPC and (**C**) decreases in PCr concentration between benign TSCs and TSCs with low- or high-grade PCa. (* *p* < 0.05 and > 0.005, ** *p* < 0.005).

Thin, precision-cut slices were prepared from cores of fresh prostate tissue containing no cancer (benign), low-grade cancer (Gleason score ≤ 3 + 4), or high-grade cancer (Gleason score ≥ 4 + 3). The tissue slices were cultured overnight on a rotating apparatus in a standard tissue culture incubator prior to placement in a 3-D tissue culture NMR-compatible bioreactor. Figure 2A shows representative ^31^P spectra from benign, low-grade cancer, and high-grade cancer TSCs in the bioreactor. TSCs were perfused in a gas-equilibrated medium in the bioreactor in order to maintain viability. Both benign and malignant TSCs demonstrated levels of β-NTP indicating good tissue viability, in agreement with the findings from LIVE/DEAD*^®^* viability/cytotoxicity assays (Figure S1). β-NTP arises from the β phosphate group of the nucleotide triphosphates and provides a measurement of tissue viability [32]. The measured β-NTP concentrations did not significantly change over the time-course of the bioreactor studies, consistent with the previously established ability of a 10-mm tissue culture bioreactor to maintain prostate TSC viability for up to 24 h [19]. As seen in the representative ^31^P spectra shown in Figure 2, the inorganic phosphate resonance (Pi) is dominated by the Pi in the buffer used in the perfusion media negating the ability to measure an intracellular pH. Similar to our findings in previous in vivo [33] and ex vivo [19] studies, an increase in the phosphomonoester region of the ^31^P spectra and a decrease in phosphocreatine (PCr) in cancer were observed (Figure 2A, red dashed lines). Quantitatively, there was a significant (*p* < 0.05) increase in the phosphocholine (PC)/glycerophosphocholine (GPC) ratio between high-grade cancer (3.89 ± 0.89) and low-grade cancer (1.94 ± 0.28) or benign tissue (1.27 ± 0.38) (Figure 2B). The concentration of PCr significantly (*p* < 0.005) decreased from 14.4 ± 2.1 nmols in benign tissue to 8.1 ± 0.6 nmols in low-grade cancer and 5.4 ± 0.7 nmols in high-grade cancer (Figure 2C). PCr levels were not significantly different between high-grade and low-grade cancer (*p* = 0.359). 

### 2.3. HP ^13^C MRS of TSCs: Gleason Grade-Dependent Increases in HP ^13^C Lactate Signal and Efflux

**Figure 3:**
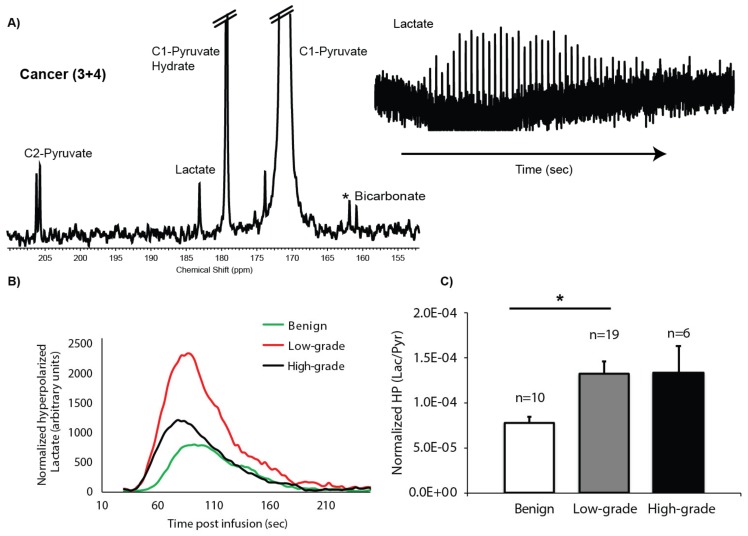
Hyperpolarized (HP) ^13^C spectroscopy of TSCs. (**A**) Representative summed ^13^C spectrum from a TSC containing 44% cancer of Gleason score 3 + 4 post-injection of HP [1-^13^C]pyruvate (left) as well as the dynamic time course of the HP [1-^13^C]lactate signal (right); (**B**) Representative HP lactate kinetics from each type of TSC (benign, low-grade cancer, or high-grade cancer), normalized to the βNTP peak; (**C**) Bar graph of normalized ratios of AUC_Lac/Pyr_ demonstrates a significant difference only between the benign and low-grade cancer TSCs (* indicates *p* < 0.05).

**Figure 4:**
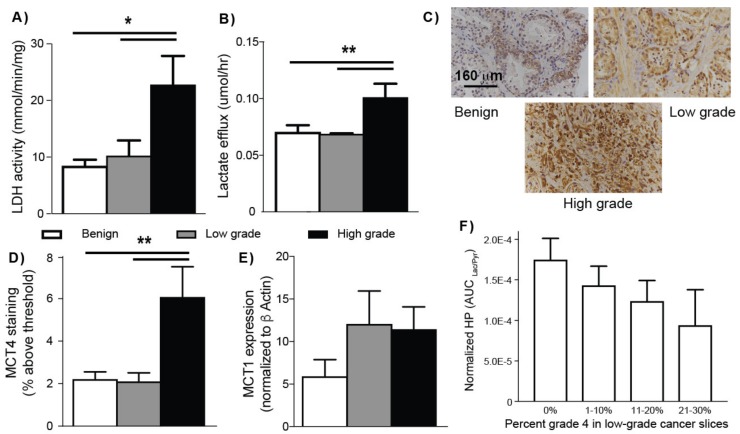
Increased lactate production and efflux in tissue slice cultures (TSCs) with high-grade cancer. (**A**) TSCs containing high-grade cancer have the highest level of LDH activity/mg of tissue in comparison to benign TSCs and TSCs with low-grade cancer; (**B**) Measurement of the [3-^13^C]lactate efflux (μmol/h) from TSCs shows that TSCs with high-grade cancer has significantly increased efflux compared to TSCs with low-grade cancer and benign TSCs; (**C**) Representative IHC staining with anti-MCT4 antibody of the three types of prostate TSCs; (**D**) Bar graph of MCT4 staining (% above threshold) demonstrated a significant increase in MCT4 in high-grade cancer compared to lower and near similar levels in the benign and low-grade cancer TSCs; (**E**) *MCT1* mRNA expression was quantified by qRT-PCR in benign (N = 5), low-grade cancer (N = 4), and high-grade cancer (N = 5) in prostate TSCs; (**F**) The hyperpolarized (HP) AUC_Lac/Pyr_ has an inverse linear relationship to the percentage of Gleason grade 4 in low-grade cancers (having primary Gleason grade 3, N = 17). This indicates a linear relationship between percentage of high-grade cancer and increased HP [1-^13^C]lactate efflux via MCT4 and implies the association of efflux with high-grade cancer; (* *p* < 0.05 and > 0.005, ** *p* < 0.005 and > 0.0005).

Dynamic data of the conversion of HP [1-^13^C]pyruvate to [1-^13^C]lactate with a good signal-to-noise ratio (SNR) were robustly obtained from 60 to 80 mg of prostate TSCs maintained in the 5-mm 3-D tissue culture NMR-compatible bioreactor, with peak HP [1-^13^C]lactate having an average SNR of 13 ± 1. The left side of Figure 3A shows a representative spectrum from TSCs containing cancer of Gleason score 3 + 4, acquired at 54 s after infusion of HP [1-^13^C]pyruvate at the time of maximum HP [1-^13^C]lactate signal. In tissue culture bioreactor studies, the HP [1-^13^C]pyruvate peak (171 ppm) is very large due to the large volume of medium in the sensitive volume of the coil. Also observed is the [1-^13^C]pyruvate hydrate peak (179.3 ppm) and the natural abundance [2-^12^C]pyruvate peak (206 ppm), which is split into a doublet by the coupling to the ^13^C labeled C-1 peak, as well as the metabolic products [1-^13^C]lactate (183.2 ppm) and, in some spectra, the ^13^C bicarbonate peak (160.8 ppm). Unfortunately, less than half of the HP ^13^C MR spectra contained a quantifiable bicarbonate peak, thereby negating the ability to determine any significant differences in the conversion of HP [1-^13^C]pyruvate to [1-^13^C]bicarbonate between the prostate tissue types investigated. On the right side of Figure 3A is a stack plot of HP [1-^13^C]lactate over time after injection of HP [1-^13^C]pyruvate. Since acquisition is started at time zero, there is a 30-s delay in the arrival of the [1-^13^C]pyruvate (Figure 3B) in the sensitive volume of the coil where the tissue chamber is located. Figure 3B shows representative dynamic time courses of HP [1-^13^C]lactate signal from benign, low-grade cancer, and high-grade cancer TSCs. At the 3 s temporal resolution of the dynamic HP ^13^C MRS acquisition, the time to maximum peak lactate was similar across the different types of prostate tissue and was on average 54 ± 1 s from the time of the first appearance of HP [1-^13^C]pyruvate. Figure 3C shows the mean HP area under the curve (AUC)_Lac/Pyr_ for benign prostate tissues (N = 10), low-grade cancer (Gleason score ≤ 3 + 4, N = 19) and high-grade cancer (Gleason score ≥ 4 + 3, N = 6). The benign TSCs had the lowest HP AUC_Lac/Pyr_ (7.81 ± 0.69 × 10^−5^), with a significant (p < 0.05) increase in the low-grade cancer HP AUC_Lac/Pyr_ (14.2 ± 1.6 × 10^−5^) which was not significantly different in high-grade cancer (13.4 ± 3 × 10^−5^). The lack of increased HP AUC_Lac/Pyr_ in high-grade cancer is due to increased [3-^13^C]lactate efflux in high-grade PCa, as shown in Figure 4, and the subsequent loss of the effluxed HP [1-^13^C]lactate in the perfusion media from the sensitive volume of the RF coil due to the continuous flow conditions employed in the bioreactor experiment, as has been validated in prior studies [34,35,36].

Consistent with the significantly increased *LDHA* mRNA expression and tumor tissue lactate concentrations observed in high-grade PCa biopsies (Figure 1), Figure 4A shows significantly increased LDH activity in TSCs with high-grade cancer (22.82 ± 5.05 mmols/min/mg) as compared to both benign TSCs (8.49 ± 1.05 mmols/min/mg) and TSCs with low-grade cancer (10.26 ± 2.68 mmols/min/mg). Interestingly, LDH activity was not significantly increased in low-grade cancer compared to benign prostate tissues in this study. The lactate efflux rate of the TSCs is easily measured in the steady state culture conditions using high resolution ^1^H NMR of the medium over time, with the addition of [3-^13^C]pyruvate. The pyruvate in the culture medium of the TSCs is taken up and converted to [3-^13^C]lactate and subsequently exported out of the cell, into the medium. Figure 4B shows that the lactate efflux rate was significantly increased in TSCs with high-grade cancer (0.100 ± 0.012 μmols/h) compared to benign TSCs (0.07 ± 0.006 μmols/h) and TSCs with low-grade cancer (0.07 ± 0.0002 μmols/h). Similar to LDH activity, lactate efflux rate was not significantly increased in TSCs with low-grade cancer compared to benign TSCs. Expression of transmembrane MCT4, the predominant transporter for lactate efflux from the cell, paralleled the [3-^13^C]lactate efflux rate. TSCs with high-grade cancer had significantly higher MCT4 expression (6 ± 1.4%) than either benign TSCs (2 ± 0.3%) or TSCs with low-grade cancer (2 ± 0.3%) (Figure 4C,D). Concurrently while the MCT1 mRNA expression, the gene that encodes for the monocarboxylate transporter predominantly responsible for the uptake of pyruvate, was increased two-fold in PCa compared to the benign TSCs (Figure 4E), there was no significant difference between low- and high-grade PCa. Moreover, HP AUC_Lac/Pyr_ had a significant (*p* = 0.028) inverse correlation (Spearman non-parametric) of r = −0.53 to the percentage of Gleason grade 4 cancer in low-grade cancer slices (primary pattern 3; Figure 4F) due to increased lactate efflux out of the cancer cells via the upregulated MCT4 expression with the prevalence of grade 4 disease (N = 17).

In summary, these observations demonstrate that the increased level, as well as efflux, of lactate in high-grade cancers are associated with increased *LDHA* mRNA expression, LDH activity, and MCT4 protein expression, respectively.

## 3. Discussion

Management of patients with localized PCa relies heavily on characteristics, such as the Gleason grade and amount of cancer, of transrectal ultrasound targeted biopsies. However, prostate biopsies suffer from large sampling errors, typically sampling only a small percentage of the prostate, and fail to accurately discriminate aggressive versus indolent disease in many patients [14]. A non-invasive imaging method that can detect potentially high-grade aggressive PCa anywhere in the prostate at diagnosis and during active surveillance could aid in the selection of patients needing treatment and assist in the selection of the most appropriate treatment. A cut point of Gleason score ≥ 4 + 3 is often used clinically for defining high-grade/aggressive PCa that requires definitive treatment [37,38,39]. This study provides the first evidence of a significant increase of tumor lactate in high-grade (Gleason score ≥ 4 + 3) as compared to low-grade (Gleason score ≤ 3 + 4) PCa, i.e., an increased Warburg effect in living patient-derived tissue slices. Furthermore, we demonstrate that the significant increase in the Warburg effect in high-grade PCa occurs in conjunction with increased lactate efflux via increased MCT4 expression. These findings can potentially provide two non-invasive imaging biomarkers for discriminating indolent from aggressive PCa.

This study reports, for the first time, a Gleason grade-dependent increase in lactate concentration and *LDHA* mRNA expression in PCa snap-frozen patient biopsies. In a prior quantitative ^1^H HR-MAS study of snap-frozen benign and PCa biopsies, a significant 2.6-fold increase in tissue lactate concentration was observed in malignant versus benign biopsies [40]. However, an insufficient number of biopsies containing cancer of varying pathologic grades was assayed in the prior study to determine if there was a significant change in lactate concentration with pathologic grade. In this study, significant increases in tissue lactate concentration were observed between benign tissue and low-grade PCa (1.4-fold), between benign tissue and high-grade cancer (4-fold), and, more importantly, between low- and high-grade PCa (2.9-fold) (Figure 1A). Furthermore, the cancer- and grade-dependent increases in lactate concentration in biopsies correlated with mRNA expression of *LDHA*, the gene that encodes the LDH enzyme subunit with the highest affinity for reduction of pyruvate to lactate. Specifically, there was a 1.6-fold increase in *LDHA* expression in high- versus low-grade PCa. *LDHA* expression levels have been previously correlated with prostate cancer progression, metastasis, and biochemical recurrence [41,42]. Unfortunately, the ability to detect tissue lactate in in vivo ^1^H MRSI studies of PCa is inhibited by large contributions from lipids that surround the prostate and whose resonances overlap the lactate resonance chemical shift [20].

The increased signal enhancement provided by HP ^13^C MR and the lack of background ^13^C signals from lipids makes it an ideal technique for assessing PCa pathologic grade in vivo based on changes in HP [1-^13^C]lactate. While a prior HP [1-^13^C]pyruvate prostate tissue slice bioreactor study demonstrated a significant increase in HP [1-^13^C]lactate in PCa relative to benign prostate tissues [19], this is the first study to investigate a sufficient number of patient-derived TSCs to provide a correlation between HP [1-^13^C]lactate and pathologic grade. Specifically, there was a 1.8-fold increase in HP [1-^13^C]lactate in TSCs with PCa (both high- and low-grade) relative to benign TSCs, similar to the prior bioreactor study [19]. Moreover, this study demonstrates that high-grade PCa is not only associated with significantly higher lactate but also rapid HP [1-^13^C]lactate efflux, as a result of high MCT4 expression, compared to low-grade PCa and benign prostate tissue. The lack of increased total HP [1-^13^C]lactate (extracellular and intracellular) signal in high- versus low-grade PCa observed in the bioreactor study is due to a 1.4-fold increase in the rate of lactate efflux as measured by [3-^13^C]pyruvate studies in high-grade PCa and subsequent loss of signal of the HP [1-^13^C]lactate in the media due to the flow conditions employed in the bioreactor experiment. This loss of HP [1-^13^C]lactate due to efflux and media flow has been validated in several prior bioreactor studies of renal cancer cells [35,36]. Further biological evidence supporting a significant increase of lactate and its efflux in high- versus low-grade PCa TSCs include: (1) Significant elevation of LDH activity in high-grade cancer TSCs (2.7- and 2.2-fold relative to benign and low-grade cancer TSCs, respectively), while the difference in LDH activity was not significant between TSCs with low-grade PCa and benign TSCs; and (2) Significant elevation of MCT4, the predominant symporter of lactate and proton efflux, in high-grade cancer TSCs (3-fold increase in high- versus low-grade PCa and benign prostate TSCs), while there was no significant difference in MCT4 expression between low-grade PCa and benign TSCs.

The significant increase in MCT4 observed in high-grade PCa TSCs is also consistent with prior publications indicating that high MCT4 expression predicts aggressive disease in many cancers [43], and specifically in PCa, where it has been shown to correlate with high-grade cancer (Gleason ≥ 7) and other clinical characteristics associated with poor prognoses, such as higher serum PSA levels at diagnosis and extension of cancer through the prostatic capsule [29,30,44]. MCT4 is a low-affinity monocarboxylate transporter that is primarily responsible for the export of lactate and its associated proton from the cell [37,38] and, like other glycolytic enzymes, is upregulated by hypoxia (unlike other isoforms of MCT) [39]. Apart from MCT4, MCT1 is the other monocarboxylate transporter expressed in PCa. MCT1 has a higher affinity (>6-fold) for pyruvate over lactate transport [45] and does not contribute to the net transport of lactate [46]. Furthermore, it has been shown that when solid tumors co-express both isoforms, inhibition of MCT1 reduces cell proliferation but does not alter lactate efflux via MCT4 [47] and confers drug resistance in hypoxia [48], suggesting a lactate-transport independent role of MCT1 promoting tumor proliferation.

Another important finding of this study is that the HP [1-^13^C]lactate has an inverse relation with the percentage of Gleason grade 4 in low-grade cancers (Figure 4F). The fact that the correlation was inverse is due to the 60% increase in [3-^13^C]lactate efflux observed in high-grade versus low-grade cancer and benign TSCs, thereby associating with the prevalence of Gleason grade 4 or higher disease. For in vivo studies, the HP [1-^13^C]lactate that effluxes out of the cell will remain predominately in the tumor microenvironment and should add rather than subtract from the HP [1-^13^C]lactate signal, providing a large increase in hyperpolarized lactate with the presence of Gleason grade 4 and 5 cancer. This supposition is consistent with prior studies involving renal cell cancer cells implanted under the renal capsule [49]. The ability to assess the percentage of Gleason grade 4 and 5 cancer in a tumor has significant implications for selection of the appropriate therapy for patients since cancers with a Gleason score of ≤ 3 + 3 are considered indolent and appropriate for active surveillance while a Gleason score of 7 or higher portends more aggressive cancer. Furthermore, the percentage of Gleason grade 4 present in the cancer impacts risk of progression [50], i.e., a Gleason score of 3 + 4 has a favorable prognosis with estimated 4-year biochemical recurrence-free survival of 82% compared to 65% for a Gleason score 4 + 3 tumor, which is more similar to the risk for a tumor of Gleason score 4 + 4 [51].

^31^P NMR spectra acquired during this prostate TSC bioreactor study demonstrated good TSC viability, as evidenced by good β-NTP levels that did not significantly change over the time-course of the bioreactor studies, consistent with the prior prostate TSC bioreactor study [19]. The ^31^P NMR spectra also demonstrated several pathologic grade-dependent changes that are consistent with prior published studies. There was a significant 3-fold increase in the ratio of the phospholipid metabolites, PC/GPC, between benign TSCs and TSCs with low-grade cancer, and a significant 1.8-fold increase in PC/GPC between low-grade and high-grade cancer. The increase in phospholipid metabolites in PCa relative to benign prostate tissue is consistent with prior in vivo transrectal ^31^P MRS studies of the in vivo human prostate [33]. The cancer grade-dependent increase of PC/GPC in this study was also consistent with a quantitative 1D and 2D ^1^H HR-MAS total correlation spectroscopy study of 49 prostate surgical samples that demonstrated that high-grade (Gleason score ≥ 4 + 3) PCa had significantly higher proliferation (Ki67 labeling) and concentrations of PC and GPC than low-grade cancers [52]. We also observed a significant 2-fold decrease in the PCr concentration between benign prostate TSCs and TSCs with low-grade cancer, with a further non-significant 1.2-fold reduction of PCr in TSCs with high-grade versus low-grade cancer. A significant reduction in PCr was observed in previous in vivo transrectal ^31^P MRS studies of the in vivo human prostate [33] and in a prior study of PCa TSCs [19]. The reduction of PCr is consistent with prior studies that revealed that creatine kinase isoenzymes that control the steady state levels of PCr are under hormonal control and are reduced in PCa due to a reduction in androgen sensitivity [53]. Unfortunately, the sensitivity of ^31^P MRS, due to its low gyromagnetic ratio, γ (γ of ^31^P ≈ 1/2 that of ^1^H), greatly limits its utility in assessing the presence and aggressiveness of prostate tumors in patients. 

The main limitation of this study is that the often small-volume and multifocal nature of PCa, particularly low-grade PCa, did not provide sufficient tissue at surgery to perform all of the necessary studies on the same tissues used in the TSC bioreactor studies. We therefore measured steady state tissue concentrations of lactate in benign, low-, and high- grade PCa from snap-frozen biopsies. Additionally, we did not have sufficient amounts of tissue to measure all of the factors that could lead to increased HP [1-^13^C]lactate in the TSC bioreactor studies. Specifically, increased lactate production in addition to higher LDH activity could also arise from increased pyruvate import via MCT1 and/or increased availability of the cofactor of LDH, nicotinamide adenine dinucleotide (NADH). We found that *MCT1* mRNA expression in cancers was two-fold higher than benign tissue similar to a prior study [19]; however, there was no significant difference between low- and high-grade cancers (Figure 4E). Additionally, there was not sufficient tissue to measure NADH. Another limitation is that lactate efflux could not be measured in the bioreactor due to the relatively small amount of HP [1-^13^C]pyruvate injected and the large reservoir of perfusion medium. Therefore, lactate efflux was measured with pathologic grade-matched TSCs not used in bioreactor studies in 2-D culture over-time using non-polarized [3-^13^C]pyruvate in the medium.

Notwithstanding these limitations, this study shows that high tissue lactate and rapid lactate efflux are dominant metabolic features of high-grade PCa. Moreover, a number of recent developments support the feasibility of clinically translating these preclinical findings to patient HP [1-^13^C]pyruvate studies. HP [1-^13^C]pyruvate has already been granted FDA approval as an investigational new drug (IND) for use in patients with PCa and has been evaluated in men undergoing a multi-parametric ^1^H MRI PCa staging exam [54]. Based in part on the results of this preclinical prostate TSC study, patient studies have been initiated to investigate the relationship between the apparent rate of HP [1-^13^C]lactate generated from [1-^13^C]pyruvate and cancer grade using whole mount step section pathology after radical prostatectomy as the standard of reference (NCT02526368). One approach for estimating lactate efflux in vivo is based on the concept that the compartmental (intra- versus extra-cellular) difference in mean free diffusion path length would be reflected by the apparent diffusion constant (ADC). While the clinical feasibility of measuring HP lactate ADC is still under development, there are numerous preclinical studies that have shown that it can be measured and used to stratify tumor aggressiveness [49,55,56,57,58,59].

## 4. Materials and Methods

### 4.1. Quantitative HR-MAS NMR Spectroscopy of Prostate Biopsies

As previously described [19], ultrasound-guided prostate biopsies were acquired from 30 patients [15 from benign regions of the peripheral zone, 11 from regions of low-grade (Gleason score ≤ 3 + 4) cancer, and 4 from regions of high-grade (Gleason score ≥ 4 + 3) cancer] after approval by the UCSF Institutional Review Board (IRB) and informed consent using an 18- gauge needle (15-mm X 1-mm cores). Biopsies were placed in individual cryovials and snap-frozen on dry ice (≤15 s) immediately after the procedure. The tissues were processed for HR–MAS NMR, as described by Tessem et al. [40]. Briefly, each biopsy was weighed prior to loading into an HR–MAS rotor with a known mass of 3.0 μL of deuterium oxide containing 0.75% weight/volume sodium-3- trimethylsilylpropionate-2,2,3,3-d4 (D_2_O + TSP). Quantitative ^1^H HR–MAS NMR was performed at 11.7 T Varian INOVA NMR with a 4-mm gHX nanoprobe (Agilent Technologies, Santa Clara, CA, USA ) at 1 °C, and 2250 Hz spin rate. Fully relaxed pulse-acquire spectra were acquired with a 2-s presaturation delay, 2-s acquisition time, 40,000 points, 20,000-Hz spectral width, and 128 transients. The data were processed and quantified using the previously published HR–QUEST technique using ERETIC, an electronic reference to access ex vivo concentrations [60], yielding absolute concentrations of lactate [40].

### 4.2. Prostate Tissue Slice Acquisition and Culture

Prostate tissues were obtained with approval by the IRB at UCSF (10-02033) and Stanford University (13895) and informed consent. Fresh tissue cores (8-mm diameter) were obtained from 37 radical prostatectomy specimens immediately following surgery from 12 regions of benign peripheral zone, 20 regions of low-grade (Gleason score ≤ 3 + 4) cancer, and 6 regions of high-grade (Gleason score ≥ 4 + 3) cancer. Clinical characteristics of the tissue donors (age, serum PSA, and stage) are provided in Table 1. Each tissue core was mounted in a Krumdieck tissue slicer (Alabama Research and Development, Munford, AL, USA) and rapidly sectioned (300- to 400-μm thickness) while immersed in chilled HEPES-buffered saline. The tissue slices were then placed on titanium grids in 6-well dishes containing Complete PFMR-4A with 50 nM of R1881 [31,61]. Following overnight culture on a rotator specifically designed for tissue slice culture (Alabama Research and Development) at 37 °C in 95% air/5% CO_2_, viability was assessed using a LIVE/DEAD^®^ Viability/Cytotoxicity assay (Invitrogen, Thermo Fisher Scientific, Waltham, MA, USA). The TSCs were then placed in the bioreactor for dynamic HP ^13^C NMR spectroscopy and subsequent immunohistochemical and biochemical assays.

### 4.3. 3D Tissue Culture NMR-Compatible Bioreactor

The micro-engineered 5-mm bioreactor is a sophisticated perfusion system, under pressure with a continuous flow of 37 °C media at 0.5 mL/min and 20% oxygen to maintain the physiologic and functional properties of tissue cultures [62]. A specially designed, precision 3D printed cartridge was used to gently restrain 4–6 tissue slices (60–80 mg) from each patient per bioreactor study in the active region of the radiofrequency (RF) coil to ensure maximal sample filling and B_o_ homogeneity.

### 4.4. NMR Spectroscopy Measurements of Prostate TSCs

^31^P spectra were obtained to monitor tissue viability and to evaluate the metabolic profile of benign and malignant prostate TSCs using a sweep width of 20 KHz, 40,000 points, and 2048 repetitions immediately preceding the HP ^13^C experiment. The phosphorous spectra were quantified using ERETIC [60], implemented on the X-channel of the NMR spectrometer. The ERETIC peak area was periodically calibrated against known concentrations of ATP standards. The metabolite peak areas were used to calculate their concentrations based on the ATP-calibrated ERETIC peak area, after accounting for the differential relaxation rates (T_1_) [36]. 

For HP ^13^C studies, [1-^13^C]pyruvic acid (14.2 M) and the trityl radical OX063 (15 mM) (GE HealthCare, New York, NY, USA) were polarized using the dissolution dynamic nuclear polarization method in the HyperSense (Oxford Instruments, Abingdon, UK) and subsequently dissolved in phosphate buffer, as previously described [35]. NMR data were acquired on a narrow bore 11.7 T Varian INOVA equipped with a 5 mm broadband probe. HP ^13^C spectra were acquired dynamically (30 degree pulses, 3 s temporal resolution for 300 s) following injection of 750 µL of the 4 mM pyruvate dissolution solution into the bioreactor with continuous flow in 90 s.

All HP ^13^C data were processed using ACD Lab with 5 Hz broadening and Lorentzian peak fitting. The HP data are presented as mean ratio of lactate to pyruvate peak area under the entire dynamic curve (AUC_Lac/Pyr_) and is computed as = where *n* is the number of dynamic time points acquired [63,64], by summing all the spectra and taking the ratio of the peak areas of lactate to pyruvate. This ratio was also normalized to nmols of β-NTP in order to correct for differences in the amount of viable tissue between bioreactor studies experiments in the active region of the RF coil and scaled by 10^-5^. Only those peaks with a signal-to-noise ratio (SNR) of at least 5 were included in the analysis.

### 4.5. Measurement of TSC Lactate Efflux Rate

Some of the tissue slices after overnight culture (and not used for the bioreactor experiments) were used to measure the rate of lactate efflux. One slice per well (placed on the titanium mesh) was incubated in Complete PFMR-4A with 50 nM of R1881 (as mentioned above) containing 25 mM [3-^13^C]pyruvate. The rate of lactate efflux from the tissue slices was evaluated by collecting the medium every 60–120 min over 8 h and replacing with fresh medium at each time point. Fully relaxed ^1^H NMR spectra were obtained for each of the sampled media with a known mass of D_2_O + TSP in an 800 MHz Bruker DRX spectrometer equipped with a cryo-cooled 5mm triple-axis heteronuclear probe using 12,288 points, sweep width of 12 KHz, and 32 repetitions. To quantify the [3-^13^C]lactate produced and exported by the tissue slices from the [3-^13^C]pyruvate taken-up from the medium, N = 4 high-grade cancer, N = 3 low-grade cancer, and N = 3 benign slices were used. The data was processed using ACD/Labs software and the J-coupled ^13^C satellite resonance areas of the lactate were quantified.

### 4.6. Immunohistochemistry (IHC)

Immediately after the bioreactor and ^1^H HR-MAS spectroscopy experiments, the tissue slices and biopsies were embedded in optimal cutting temperature (OCT) compound and sectioned at 5-μm for immunohistochemical staining. As described in previous studies [19], each section was stained with hematoxylin and eosin (H&E). An experienced prostate cancer pathologist reviewed each section on two separate days and determined the Gleason score [65] in addition to the percentage of benign epithelium, stroma, and cancer cells in each tissue section based on the H&E staining (Table 1). The tissues were grouped into 3 cohorts: benign (no cancer present), low-grade cancer (Gleason score ≤3 + 4) and high-grade cancer (Gleason score ≥ 4 + 3). Additionally, the percentage of cancer comprised of Gleason grade 4 was assessed for each low-grade cancer tissue slice. The pathologist also marked regions of benign epithelium and cancer on the sections which were used as guidelines to scrape the respective tissue from the OCT embedding for further RNA extraction in case of biopsy and LDH activity for the tissue slices. Furthermore, the tissue slices were stained with anti-MCT4 antibody (rabbit polyclonal primary antibody SC-376140 from Santa Cruz Biotechnology Inc., Dallas, TX, USA) in 1:100 dilution at 4 °C overnight, followed by incubation with rabbit secondary antibody/ HRP at 1:200 dilution for 20 min at room temperature as before [49], to detect MCT4 at the cell membrane.

### 4.7. Image Analysis of MCT4 IHC

H&E- and MCT4-stained tissue sections were imaged with a Nikon 6D microscope under bright-field illumination using a 0.15 µm^2^ in-plane resolution. The images were processed using Matlab’s color thresholding toolbox. First, the image in the hue-saturation-value (HSV) color space was thresholded with the hue values on the interval of [0,0.176] to capture the brown, the interval of [0.45,1.000] for the saturation values, and the intensity values in the interval of [0,0.535] to capture the darkest brown staining for expression of MCT4 on the cell membrane. Next, image regions focused on the cancer cells were analyzed from the resulting binary image, and the total pixel count of the detected regions was quantified as a % of the entire area. Two to four focal regions per case were imaged and analyzed for MCT4 expression and the mean values are reported.

### 4.8. Biochemical and Molecular Assays

Tissue slices were homogenized after 24 h of culture and assayed for ATP content. ATP concentration was measured in the TSCs using the StayBrite ATP bioluminescence kit (BioVsion Inc., Milpitas, CA, USA) and the Veritas microplate luminometer (Turner Biosystems, Sunnyvale, CA, USA).

Frozen tissue (3–5 mg) was homogenized using the Tissuelyser LT (Qiagen, Hilden, Germany) in lysis buffer (Cell Signaling, Danvers, MA, USA) for measurement of LDH activity. The enzyme activity was quantified using a standard calorimetric method of measuring the initial enzyme velocity as a linear decrease in absorbance of NADH using varying concentrations of pyruvate added to homogenized tissue lysates [19].

Total RNA was extracted from tissues with an RNAeasy kit (Qiagen) and qRT-PCR was used to measure the expression of the *LDHA* gene utilizing primers purchased from Applied Biosystems (Hs01378790_g1). As described previously [19], reverse transcription using an iScript cDNA Synthesis kit (BioRad Laboratories, Hercules, CA, USA) was performed and the cDNA generated was utilized for PCR in triplicate with TaqMan chemistry on the ABI 7900HT (Applied Biosystems, Thermo Fisher Scientific, Waltham, MA, USA). The mRNA expression of *LDHA* was calculated relative to the housekeeping gene *ACTB* (Amplicon:TACGCCAACACAGTGCTGTCTGGCGGCACCACCATGTACCCTGGCATTGCCGACAGGATGCAGAAGGAGATCACTGCCCTGGCACCCAGCACAATGAAGATCAAGATCATTGCTCCTCCTGAGCGCAAGTACTCCGTGTGGATCGGCGGC, Forward: TACGCCAACACAGTGCTGTCT, Reverse: GCCGATCCACACGGAGTACT, Probe: ATCAAGATCATTGCTCCTCCTGAGCGC).

### 4.9. Statistics

Statistical analyses were performed using PRISM (GraphPad Software, San diego, CA, USA). For multiple comparisons, one-way ANOVA was used with a 2-stage linear step-up procedure of Benjamini, Krieger, and Yekutieli for post-hoc tests using a false discovery rate, Q < 0.05. All data are represented as mean ± standard error and significant (*p* < 0.05) differences between the groups are denoted by an asterisk (* *p* < 0.05 and > 0.005, ** for *p* < 0.005 and > 0.0005, and *** for *p* < 0.0005). The relationship between HP AUC_lac/pyr_ and percentage of Grade 4 cancer cells was assessed by the nonparametric Spearman correlation coefficient (*r*).

## 5. Conclusions

In summary, consistent with prior preclinical HP ^13^C MR publications involving murine models, the progression from low- to high-grade human PCa resulted in a significant increase in lactate (i.e., the Warburg effect) and an associated increase in LDH activity in living prostate tissue cultures. Another important finding of this study is the significant increase in MCT4-facilitated lactate efflux as a clear distinguishing factor for the presence of Gleason grade 4 PCa. Moreover, the lack of a significant increase in LDH activity and lactate efflux in low-grade PCa versus benign tissue suggests that HP [1-^13^C]pyruvate MRI can be used to image the presence of high-grade cancer in patients at the time of diagnosis, or during disease progression while on active surveillance. This hypothesis is being tested in a recently initiated HP [1-^13^C]pyruvate MRI study of patients with early-stage/indolent PCa selecting or on “active surveillance” as their clinical management course and receiving MRI/TRUS fusion biopsy as the pathologic standard of reference (NCT03933670).

## Figures and Tables

**Table 1:** Patient Characteristics.

ID	Age	Pre-op PSA ng/mL	Stage	Gleason Score	% Cancer Cells	% Epithelial Cells	% Stroma
1	55	5.2	pT2c pN0	3 + 3	7	37	57
2	53	5	pT2a	3 + 4	53	21	25
3	63	6.3	pT2c pN0	3 + 4	19	27	54
4	56	7.6	pT2a	N	--	40	60
5	50	4.5	pT2c	N	--	40	60
6	65	7.1	pT2c	N	--	46	53
7	54	6.9	pT2c	3 + 4	84	2	15
8	67	3.1	pT2b	N	--	36	65
9	60	6.6	pT2c	N	--	45	53
10	71	6.9	pT3b	3 + 4	27	36	38
11	71	6.9	pT3b	4 + 5	33	36	31
12	55	5.4	pT3a	4 + 5	31	29	41
13	55	5.4	pT3a	N	--	37	63
14	61	6.8	pT2c	3 + 4	31	33	36
15	54	4.7	pT2c	3 + 4	49	33	19
16	63	4.1	pT3a	3 + 4	44	16	41
17	71	5	pT3b pN0	3 + 4	11	48	43
18	52	15.8	pT3b	3 + 3	26	43	31
19	52	6.3	pT3a	3 + 4	20	20	60
20	71	8.1	pT3a	3 + 4	48	16	36
21	51	11.1	pT3b pN0	3 + 3	6	19	74
22	67	4.2	pT2c	N	--	28	72
23	67	3.5	pT2c pN0	3 + 4	16	32	52
24	72	5.8	pT3a	N	--	20	80
25	69	5	pT2c	N	--	44	56
26	61	7.87	pT2c	3 + 3	42	27	32
27	64	24.37	pT3a	4 + 5	40	18	43
30	65	2.11	pT3a pN0	3 + 4	2	41	58
29	65	2.56	pT3a	N	--	40	61
31	56	7.5	pT2c	N	--	34	67
32	56	4.5	pT2c	N	--	47	53
33	58	13.9	pT3a pN0	3 + 3	1	34	65
34	66	20.6	pT3b	4 + 5	31	8	61
35	69	13.8	pT3b NX	4 + 4	8	23	68
36	71	7.2	pT3a	3 + 4	35	25	40
37	61	5.6	pT2c	4 + 3	48	3	51
38	68	8.4	pT3a pN0	3 + 4	18	33	49
39	71	5.9	pT3a pN0	3 + 4	11	35	54

## Supplementary Materials

The following are available online at https://www.mdpi.com/2072-6694/12/3/537/s1, Figure S1: Live/Dead assay for measuring tissue viability.
